# Advances in Cardiovascular Multimodality Imaging in Patients with Marfan Syndrome

**DOI:** 10.3390/diagnostics15020172

**Published:** 2025-01-14

**Authors:** Marco Alfonso Perrone, Sara Moscatelli, Giulia Guglielmi, Francesco Bianco, Deborah Cappelletti, Amedeo Pellizzon, Andrea Baggiano, Enrico Emilio Diviggiano, Maria Ricci, Pier Paolo Bassareo, Akshyaya Pradhan, Giulia Elena Mandoli, Andrea Cimini, Giuseppe Caminiti

**Affiliations:** 1 Division of Cardiology and CardioLab, Department of Clinical Sciences and Translational Medicine, University of Rome Tor Vergata, 00133 Rome, Italy; 2 Clinical Pathways and Epidemiology Unit, Bambino Gesù Children’s Hospital IRCCS, 00165 Rome, Italy; 3 Centre for Inherited Cardiovascular Diseases, Great Ormond Street Hospital, London WC1N 3JH, UK; sara.moscatelli@gosh.nhs.uk; 4 Institute of Cardiovascular Sciences, University College London, London WC1E 6BT, UK; 5 Paediatric Cardiology Unit, Royal Brompton Hospital, London SW36NP, UK; 6 Department of Clinical Sciences and Community Health, University of Milan, 20122 Milan, Italy; giulia-guglielmi@live.it; 7 Department of Cardiovascular Sciences, AOU Ospedali Riuniti, 60126 Ancona, Italy; francesco.bianco@ospedaliriuniti.marche.it; 8 Department of Pediatrics, Marche Polytechnic University of Ancona, 60121 Ancona, Italy; cappelletti.deborah@gmail.com; 9 Department of Perioperative Cardiology and Cardiovascular Imaging, Centro Cardiologico Monzino IRCCS, 20138 Milan, Italy; amedeo.pellizon@cardiologicomonzino.it (A.P.); andrea.baggiano@cardiologicomonzino.it (A.B.); 10 Division of Cardiology, Department of Medical Biotechnologies, University of Siena, 53100 Siena, Italy; e.diviggiano@student.unisi.it (E.E.D.); giulia.mandoli@unisi.it (G.E.M.); 11 Nuclear Medicine Unit, Cardarelli Hospital, 86100 Campobasso, Italy; maria.ricci28@gmail.com; 12 School of Medicine, University College of Dublin, Mater Misericordiae University Hospital, D07 R2WY Dublin, Ireland; piercard@inwind.it; 13 Department of Cardiology, King George’s Medical University, Lucknow 226003, India; akshyaya33@gmail.com; 14 Nuclear Medicine Unit, St. Salvatore Hospital, 67100 L’Aquila, Italy; andreacimini86@yahoo.it; 15 Department of Human Science and Promotion of Quality of Life, San Raffaele Open University, 00163 Rome, Italy; giuseppe.caminiti@uniroma5.it; 16 Cardiology Rehabilitation Unit, IRCCS San Raffaele, 00163 Rome, Italy

**Keywords:** Marfan syndrome, cardiovascular multimodality imaging, cardiovascular magnetic resonance, computed tomography, echocardiography, nuclear imaging

## Abstract

Marfan syndrome (MFS) is a genetic disorder affecting connective tissue, often leading to cardiovascular complications such as aortic aneurysms and mitral valve prolapse. Cardiovascular multimodality imaging plays a crucial role in the diagnosis, monitoring, and management of MFS patients. This review explores the advancements in echocardiography, cardiovascular magnetic resonance (CMR), cardiac computed tomography (CCT), and nuclear medicine techniques in MFS. Echocardiography remains the first-line tool, essential for assessing aortic root, mitral valve abnormalities, and cardiac function. CMR provides detailed anatomical and functional assessments without radiation exposure, making it ideal for long-term follow-up. CT offers high-resolution imaging of the aorta, crucial for surgical planning, despite its ionizing radiation. Emerging nuclear medicine techniques, though less common, show promise in evaluating myocardial involvement and inflammatory conditions. This review underscores the importance of a comprehensive imaging approach to improve outcomes and guide interventions in MFS patients. It also introduces novel aspects of multimodality approaches, emphasizing their impact on early detection and management of cardiovascular complications in MFS.

## 1. Introduction

Marfan syndrome (MFS) is an autosomal dominant connective tissue disorder characterized by mutations in the FBN1 gene encoding fibrillin-1. This condition affects multiple organ systems, with cardiovascular manifestations posing the greatest threat to patient survival. The most serious complications include aortic aneurysm, aortic dissection, and mitral valve prolapse, necessitating meticulous cardiovascular monitoring [1,2].

The diagnosis of Marfan syndrome is based on well-established clinical criteria, specifically the Ghent nosology, which was developed to ensure precise identification of the syndrome and to enhance patient management and genetic counseling [1,2,3].

To mitigate the risk of premature or missed diagnoses, an international consortium of experts conducted a revision of these criteria in 2010, with the updated guidelines published in the Journal of Medical Genetics. The revised criteria emphasize the cardiovascular manifestations of Marfan syndrome, recognizing aortic root aneurysm and ectopia lentis (lens dislocation) as cardinal features of the disorder.

In the absence of a known family history, the concurrent presence of both aortic root aneurysm and ectopia lentis is sufficient for a definitive diagnosis of Marfan syndrome. However, if one of these cardinal features is absent, the diagnosis requires the presence of either a pathogenic FBN1 mutation or a positive systemic score (Table 1).

Genetic testing is often utilized in ambiguous cases to support the diagnosis. While the implementation of these revised criteria may prolong the time required for a definitive diagnosis, it reduces the likelihood of premature or missed diagnoses. Additionally, it supports a global discourse on the associated risks and guides the development of follow-up and management strategies [1,2].

Recent advancements in imaging technologies have revolutionized the management of MFS. Echocardiography, cardiovascular magnetic resonance (CMR), cardiac computed tomography (CCT), and nuclear medicine techniques each offer unique advantages in diagnosing and monitoring these patients [3]. However, significant gaps remain in understanding how to optimize the integration of these modalities for comprehensive diagnostic and monitoring strategies in MFS. This review aims to bridge this gap by providing a detailed synthesis of the latest advancements in imaging technologies and their roles in personalized care for MFS patients.

## 2. The Use of Echocardiography in Marfan Syndrome

Echocardiography represents a first-line tool in patients with MFS and it is strongly recommended after the first diagnosis and regularly in follow-up, as well as in pediatric populations. A complete echocardiographic examination is needed even if the most common related abnormalities include ascending aorta and pulmonary trunk enlargement and mitral valve prolapse [4,5].

### 2.1. Aortic Root and Proximal Ascending Aorta Dilatation

**Figure 1:**
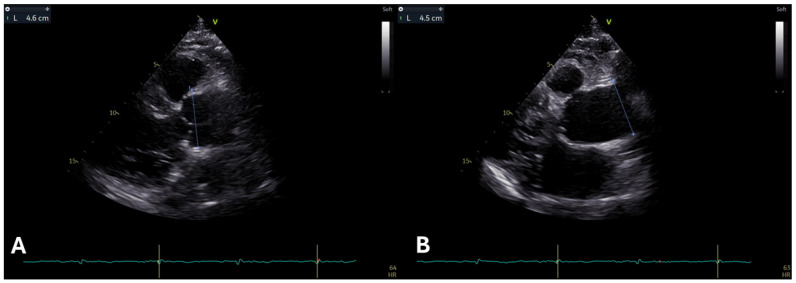
(**A**) A long parasternal view in 2D transthoracic echocardiography of a young adult reveals severe dilatation of the aortic root, displaying the characteristic “onion shape”. (**B**) A focused long parasternal view in 2D transthoracic echocardiography of the same patient demonstrates an aneurysm of the ascending aorta.

Cardiovascular adverse events remain the most common cause of death in MFS. Dilatation of the aortic root carries the highest mortality risk due to possible aortic dissection [6]. The revised Ghent nosology (2010) emphasizes the relevance of dilated aortic root [1] as a marker of MFS, being a highly specific characteristic of the disease. The distinctive pattern of this dilation, often described as an “onion shape” (see Figure 1A) is unique to MFS [4].

Transthoracic echocardiography (TTE) is the easiest and most commonly available technique to screen for aortic root enlargement. According to international recommendations, aortic root and ascending aorta should be measured at four different sections: valve annulus, sinuses of Valsalva, sino-tubular junction, and proximal ascending aorta [7]. Measurement of the aortic sinuses may inadvertently incorporate a dilated proximal coronary artery, which is also often observed in MFS, leading to potential measurement inaccuracies [8,9]. Conventionally, the ascending aorta is measured by echocardiography from the leading edge to the leading edge. Regarding sinuses of Valsalva, in patients up to 15 years of age, it has been suggested to use an inner-to-inner edge approach during systole (open aortic valve), while a leading edge-to-leading edge measurement during diastole (closed aortic valve) should be used in patients over 15 years of age and adults [10]. Two-dimensional aortic diameter measurements are preferable over M-mode measurements thanks to lower possible underestimation because of cardiac translational movement with respiration. The indexing of aortic measurements for body surface area (BSA) is of paramount importance. There are several websites offering online tools for the calculation of aortic Z-score. A Z-score ≥ 2 is considered positive for MFS [4,11,12].

MFS patients with aortic aneurysms (see Figure 1B) and those experiencing progressive aortic enlargement are at heightened risk for life-threatening events including aortic rupture and dissection and should be closely monitored [13]. The decision to undergo surgery primarily depends on the size of the aorta, guided by the natural progression of the condition and a balance between the risks of potentially adverse events versus elective surgical intervention and possible complications. In MFS adult individuals, surgery is typically recommended when the maximum diameter of the aorta reaches or exceeds 50 mm [14]. For patients with added risk factors, such as a family history of aortic dissection, aortic growth rate ≥ 3 mm per year (confirmed by consistent measurements using multiple techniques), significant aortic valve regurgitation, or plans for pregnancy, surgery might be recommended at a lower aortic diameter threshold of 45 mm [3,15].

The bicuspid aortic valve (BAV) can be found in the MFS population at a similar frequency to the general population [3,15]. In the case of a dilated aortic root highly suspected to be MFS, the BAV should be accurately screened. In the case of a poor transthoracic acoustic window, transesophageal echocardiography (TEE) is a valid alternative for accurate aortic root assessment and aortic valve morphology.

Aortic root dilatation occurs early in MFS, with 35% of patients already affected at the age of 5 and the 68% by 19 years of age. However, aorta can dilate ever late in life so long-life echocardiographic monitoring is needed in each MFS patient [16].

Several other conditions may exhibit similar echocardiographic findings. In the case of suspected connective diseases, other typical systemic signs might help in differential diagnosis with MFS. For example, a bifid uvula is characteristic of Loeys-Dietz syndrome, while translucent or lax skin is indicative of Ehlers-Danlos syndrome [4].

### 2.2. Mitral Valve

**Figure 2:**
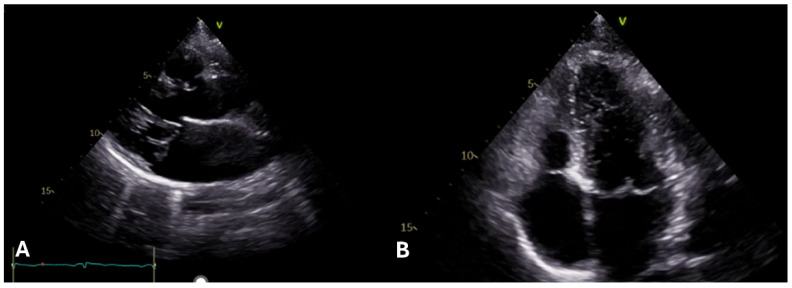
(**A**,**B**) 2D transthoracic echocardiography (TTE) images of a young adult with Marfan syndrome. Image (**A**) displays mitral valve prolapse in the long parasternal axis view, while image (**B**) shows the mitral valve prolapse in the four-chamber view.

Another common feature of MFS is an abnormal structure and function of the mitral valve. Mitral valve prolapse (MVP, see Figure 2A,B) is the second most common cardiac defect in MFS.

The occurrence of MVP ranges from 28% to 91% according to different registries [17,18,19]. Although MVP can be completely asymptomatic for a long time, it can suddenly reveal with an arrhythmic profile, in which imaging could help stratify even asymptomatic patients [20], and/or with a hemodynamic presentation, such as an acute severe mitral regurgitation (MR) when the redundant and elongated typical chordae tendineae rupture. This problem is related to the left ventricular (LV) enlargement and the degeneration of valve tissues as a consequence of extracellular matrix abnormalities [17,19]. In patients with MFS, the likelihood of MVP increases with age [18]. Echocardiographic criteria used to diagnose MVP include the prolapse of one or both leaflets by 2 mm or more beyond the long-axis annular plane, along with the thickening of the leaflets [19].

**Figure 3:**
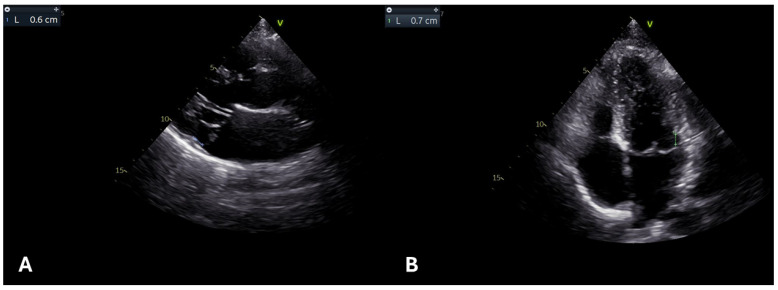
(**A**,**B**) show mitral annular disjunction (MAD) in 2D transthoracic echocardiography (TTE) of a young patient with Marfan syndrome, respectively, captured in the long parasternal view and the four-chamber view. The MAD measures 0.6 cm and 0.7 cm, respectively.

Mitral annular disjunction (MAD) was found in around 34% of patients diagnosed with MFS (see Figure 3A,B) and was linked to an increased risk of sustained ventricular tachycardia, sudden cardiac death, and a higher burden of premature ventricular contractions [20,21,22]. The characteristic curling motion of the lateral wall is a defining feature of MAD [12]. The presence and extent of MAD were closely linked to both the occurrence and severity of MR. The dimension of MAD was unaffected by age or BSA and remained stable over time. Further investigation is needed to ascertain if the severity of MAD correlating with the necessity for prophylactic aortic surgery [21].

In MFS, a more extended MAD can be present. MAD increases along with alterations in the mitral annular plane and dynamics, including paradoxical systolic expansion and flattening. These changes affect mitral coaptation, leading to suboptimal mitral valve geometry. The severity of MR could correlate with the extent of MAD. Patients with MFS and significant MAD may need closer clinical and Holter monitoring [23,24,25].

### 2.3. Pulmonary Artery

Pulmonary artery dilation, especially at the arterial root, is frequent in MFS. Although rare, pulmonary artery dissection may be gaining importance as MFS patients live longer [5,26,27]. According to De Backer et al., a cutoff value of ≥23 mm specifically defined pulmonary artery dilation in MFS [28]. According to the population cohort study by Nollen et al., 76% of Marfan patients exhibited an enlarged pulmonary artery at the bifurcation, and 74% had an enlarged pulmonary artery root [27]. However, this reference value lacked specificity for MFS is not related to the rare complications associated with pulmonary artery enlargement and was not included in the 2010 revised Ghent nosology.

### 2.4. Marfan Cardiomyopathy

In 1993, the term “Marfan cardiomyopathy” was coined by Hetzer and colleagues in response to the higher described prevalence of cardiomyopathy among MFS patients compared to the general population [29].

Studies indicated that increased left ventricular (LV) dimensions were observed in 7% to 68% of cases (depending on the criteria and the group studied), along with mildly reduced LV systolic function found in about 10% of the patients [30,31]. Multiple studies using echocardiography and CMR have shown mild diastolic function impairment in both adults and children with MFS. This impairment is believed to result from reduced elastic recoil due to decreased ventricular compliance and active myocardial relaxation, linked to connective tissue alterations [29,30,31,32,33,34,35,36].

In 2006, De Backer et al. found that MFS patients had a lower ejection fraction, higher indexed end-systolic volume, and reduced peak systolic velocities [34]. These findings were later confirmed by two larger studies [35,37]. A reduced LV ejection fraction does not relate with age, gender, indexed aortic dimensions, or the presence of mitral valve issues in MFS. This implies that the impairment of ventricular function is possibly linked to underlying connective tissue abnormalities.

Studies employing strain and strain rate imaging techniques to evaluate and measure alterations in global and regional contractile function have validated the results obtained from CMR, thus enhancing the diagnostic capabilities of conventional echocardiography [35,38,39,40,41].

### 2.5. Right Ventricle (RV) and Atria

In a study by Kiotsekoglou et al., notable distinctions were observed in tricuspid annular plane systolic excursion, rate of pressure rise (dp/dt), and pulsed tissue Doppler imaging (TDI) early filling measurements acquired over the lateral tricuspid valve edge, indicating impaired right ventricular (RV) function in MFS. Furthermore, signs of atrial involvement were apparent through diminished contractile, reservoir, and conduit function parameters for both atria [42,43].

LV and RV ejection fractions were strongly correlated, indicating a more emphasized interdependence between the two. However, the RV tends to be less affected, likely due to the higher workload endured by the LV [35,44,45].

## 3. The Role of Cardiovascular Magnetic Resonance in Marfan Syndrome

CMR is an essential tool in the management of MFS, which is commonly associated with progressive aortic dilation, aneurysms, and dissections. These cardiovascular complications make CMR particularly valuable, as it can assess the entire aorta, from the ascending to the abdominal portions, along with the aortic arch and descending aorta, all in a single imaging session [46,47]. Beyond aortic pathology, CMR provides comprehensive information regarding myocardial and valve function, pulmonary and peripheral arteries, as well as vascular hemodynamic, all without the use of ionizing radiation. Unlike echocardiography, CMR is not limited by poor acoustic windows, which is especially useful in MFS patients, who often exhibit musculoskeletal deformities due to their condition [46,47,48].

Therefore, CMR is the favored imaging method for serial evaluation of aortic aneurysms, especially in this young cohort of patients. It is, however, not recommended in the case of an unstable clinical context [49].

**Figure 4:**
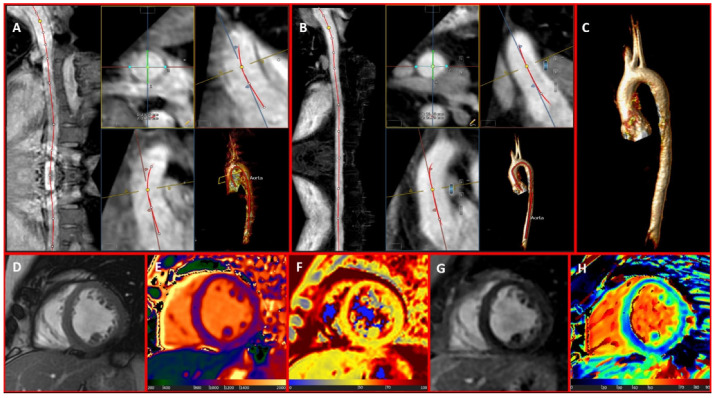
A 16-year-old girl with Marfan syndrome assessed with CMR. Box (**A**) and Box (**B**) show aortic assessments using two different sequences: Box A shows the 3D balanced steady-state free precession (bSSFP) sequence, while Box B shows the contrast-enhanced MR angiography (CEMRA) sequence. Both techniques provide comparable measurements of aortic diameters at the level of the mean pulmonary artery. Box (**C**) shows magnification of 3D volume rendering of aorta assessed with CEMRA. The patient was characterized by normal biventricular size, wall thickness, and systolic function (assessed by bSSFP sequences, Box (**D**) and by normal tissue characterization at T1 mapping Box (**E**), T2 mapping Box (**F**), LGE Box (**G**) and ECV Box (**H**) sequences).

Incorporating CMR into the routine assessment of MFS serves as a “one-stop-shop” for comprehensive cardiovascular evaluation, aiding in risk stratification, guiding therapeutic interventions, and facilitating long-term surveillance, even after surgical repair (Figure 4).

For evaluating the MFS aortic disease, CMR offers a diverse array of acquisition and reprocessing tools. Two-dimensional breath-hold steady-state free precession imaging (SSFP) cine images are useful to provide anatomical and dynamic information about aorta (including measurements), biventricular function, and valve visual assessment [47,48,49]. Adding phase contrast to the protocol is relevant in the case of valve regurgitation, especially when MV prolapse or bicuspid aortic valve are associated. Moreover, analysis of flow is important in the case of chronic aortic dissection to detect the entry and re-entry point between the true and false lumen, and to differentiate between them. The false lumen is generally bigger, lacks pulsatility, and has reduced or absent anterograde flow. Identifying the false lumen and its relationship with arising branching vessels is paramount to detect possible organ hypoperfusion and plan subsequent intervention [48,49].

Complications of aortic wall in MFS can also be detected by 2D turbo spin echo (SE) sequences, or black blood imaging techniques, which suppress the blood signal, and enhance the visualization of the vessel wall. Adding T2 weight allows for the detection of specific pathological changes, e.g., aortic inflammation/aortitis [50].

In the case of aortic dissection, intramural hematoma (IMH), or vessel tortuosity, contrast-enhanced MR angiography (CE-MRA) can be performed. In patients who cannot receive gadolinium contrast, an alternative method is the 3D-navigated SSFP, which however prolongs the acquisition time and provides suboptimal results in the case of arrhythmias [49,50].

Aortic root and proximal ascending aorta dimensions can be generally measured in 2D-SSFP (coronal, and sagittal LVOT, and axial plane) or angiographic sequences. Despite variations in measurement positions (e.g., sinus–sinus vs. sinus–commissure) and analysis strategies (such as cardiac phase selection and inclusion/exclusion of the aortic wall), consistency in the method is essential. Therefore, detailed reporting of the measurement techniques is mandatory, as it allows for maximal reproducibility in serial studies during surveillance [50,51].

Biomechanical aortic properties can also be analyzed. MRI 3D-cine time resolved phase-contrast technique (also known as 4D Flow) allows for the detailed visualization and quantification of blood flow dynamics within the vessel, including parameters like aortic stiffness, pulse wave velocity (PWV), aortic distensibility (AD), wall shear stress (WSS), or turbulent kinetic energy [51,52]. PWV measures the speed at which blood pressure waves move through the aorta, thus being a surrogate for aortic stiffness, which predisposes to aortic dilation and dissection. Typically, this parameter is elevated in MFS patients and represents a critical predictor of adverse cardiovascular events. AD, on the other hand, measures the aortic ability to expand and contract with each heartbeat and is reduced in MFS patients [53]. Four-dimensional flow technique can therefore help clinicians to detect subtle changes in aortic morphology and hemodynamics, reducing the risk of catastrophic events by facilitating early surgical or medical intervention.

Apart from the arterial evaluation, CMR is relevant in MFS to assess cardiac function. In fact, this condition is associated with a form of cardiomyopathy, which can lead to myocardial impairment [30,41,54,55,56,57]. A recent multicenter retrospective study on 241 patients with MFS showed that the presence of intrinsic myocardial dysfunction was confirmed at a young age [58]. CMR, in particular when combined with feature-tracking analyses, is able to anticipate the diagnosis of MFS cardiomyopathy and monitor its progression [31,59,60,61,62].

Moreover, MFS can be associated with an increased risk of supraventricular and ventricular arrhythmias in both adults and children [35,63,64,65], and in specific settings this prevalence has been related to mutations on exons 24–32 of FBN1 gene [66]. CE-MRA can offer precise cardiac tissue characterization, pinpointing LGE regions at a potential arrhythmogenic risk [62]. Notably, dedicated post-processing software can accurately characterize the scar, describing its size, transmurality, and composition. In the case of planned electrophysiology procedures, this model can be integrated with electro-anatomical maps, resulting in the increased success of ventricular tachycardia (VT) ablation procedures [66]. Finally, arrhythmogenic risk in MFS can also be increased in the case of MAD, which is readily identifiable through cine balanced SSFP (b-SSFP) sequences in CMR [65].

A special consideration for CMR in MFS lies in the skeletal alterations which are commonly observed, and that lead to significant impact on the heart contractile function, particularly on the right ventricle (RV). Dedicated balanced turbo field echo CMR sequences allow for the quantification of chest wall deformities using the Haller index (HI). A recent retrospective study used CMR imaging and feature-tracking strain analysis to investigate the relationship between cardiac systolic dysfunction and chest wall deformities in pediatric MFS. A high HI correlated with a more significant impairment of the cardiac function, is expressed by a reduced RV volume, ejection fraction (EF), and diminished biventricular GLS [60,61].

In conclusion, CMR has a relevant role in the evaluation of MFS patients. Not only does it provide crucial insights into aortic pathology, but it also helps assess myocardial function, brings awareness to potential arrhythmias, and evaluates skeletal-related cardiac dysfunction. By providing detailed information on both aortic disease and non-aortic cardiovascular complications, CMR helps guide timing and nature of interventions, ultimately improving patient management and long-term prognosis.

## 4. Computed Tomography Uses in Individuals with Marfan Syndrome

**Figure 5:**
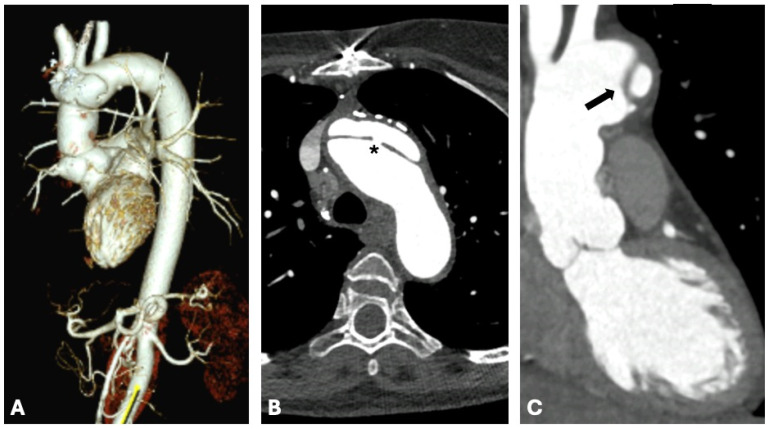
A 34-year-old woman with Marfan syndrome who underwent emergency CT examination on suspicion of aortic dissection. The asterisk and arrow panels (**B**,**C**) show the breach and the false lumen, respectively. Panel (**A**) shows the 3D reconstruction in volume rendering.

Computed tomography (CT) plays a crucial role in the diagnostic workflow for MFS, thanks to its significant advantages. These include widespread availability, excellent reproducibility, brief acquisition times, and the ability to simultaneously provide detailed images of the entire aorta—including the valve, arch, ascending, and descending portions. These features make CT extremely effective not only for identifying the most critical complications such as dissection (Figure 5), but also for complementing other imaging methods like transthoracic echocardiography (TTE) and cardiac magnetic resonance (CMR). Consequently, CT is also vital for ongoing monitoring and follow-up in patients with MFS [66,67,68].

CT protocols of acquisition should always be tailored to the clinical setting and patients. Whenever is possible, venous access is preferred on the right antecubital vein. In addition, the CT scan protocols for the aorta routinely comprise a non-contrast, an arterial, and a late scan phase. The non-contrast phase permits the assessment of aorta calcifications, mostly in adults, intramural hematoma visualization, and previous surgical prothesis or abandoned materials, where present. The arterial phase contributes to outline the aortic lumen at every segment, and the late phase may depict contrast leakage in the context of aortic dissections or previous endovascular prothesis [69,70,71].

In neonates and children, iodinated contrast agents can be injected at 2 mL/kg doses for a maximum of 100 mL of contrast amount. The dosage can be augmented at 2 mL/s in children with concomitant large intracardiac communications (i.e., large ventricular or atrial septal defects). In adults, a 1.5 mL/kg dosage is largely utilized at an infusion rate of 3–4 mL/s [70].

The acquisitions can be ECG-triggered or non-triggered. If the acquisition is triggered, the scan delay can be decided from the operator with the bolus-tracking technique. In pediatrics, the region of interest (ROI) is sited in the left ventricle at a threshold attenuation of 200 Hounsfield units (HU). In adults, the ROI is positioned in the ascending aorta, and the attenuation threshold of 140–180 HU. During synchronization, the isovolumic data can be acquired through the entire cardiac cycle and retrospectively reconstructed with an ECG-gated spiral acquisition, principally recommended in emergency settings (i.e., acute aortic dissections). When a dose reduction in radiation protocol is applied, an ECG-triggered axial (sequential) acquisition can be utilized, but the latter is more prone to heart rate irregularities and artifacts [50,67,71].

Given the wide variability of methos available for aortic imaging, it is important to standardize the assessment in order achieve accurate measurements. In addition, recommendations for the evaluation of aortic diameters are also crucial for the MFS diagnosis, disease progression estimation, and most importantly to guide surgery or medical therapy [66,72,73].

A recent report from Beetz N. L. and colleagues sought to compare CT and TTE aortic measurement for the initial work-up and for the detection of progressive aortic enlargement in a 2-year follow-up. Measurements of the differences outside the acceptable clinical limit of agreement were most frequently observed for the ascending aorta when comparing CT and TTE in MFS. In fact, despite the latter measurements showing a good correlation, the frequency of measurement differences outside the acceptable clinical limit of agreement was high, with TTE that systematically overestimates the aortic diameters. Consequently, the authors concluded that CT may be preferred for aortic monitoring patients with MFS and related disorders, while TTE remains an indispensable imaging instrument that provides additional information not available with CT [74].

The European Society of Cardiology proposed some general rules that can be applied to overcome some biased measurements of the aorta diameters. First, all the aorta measurements of diameters should be made in diastole, as systole is accompanied by a 2 mm aortic expansion [68].

The physical thickness of the aortic wall is approximately 1 mm as measured by CT. Because of the axial resolution of ultrasound, the thickness of the aortic wall is falsely increased by 1–2 additional millimeters. Subsequently, echocardiographic measurements of the aortic diameters using the inner-to-inner convention systematically underestimates the aorta dimensions when compared with CT. This technical artifact occurs at every aortic segment and should be considered when using echocardiography. Thus, echo measurements should be made utilizing the leading-to-leading edge method. In fact, an optimal agreement in leading-edge echocardiographic measurements with the inner-to-inner CT/CMR measurement in end-diastole has been demonstrated. Additionally, most of the echocardiographic studies that demonstrated the benefits of MFS’s prophylactic surgery were performed using this convention. Finally, when the aortic wall is thickened due to the presence of aneurisms or intramural hematomas, outer-to-outer diameters, that include walls, should also be reported [68,69,73].

The latest CT technologies overcome these limitations; in fact, dual-source, wide-detector, and photon counting (PC) CT scanners are able to achieve the entire thoracic aorta acquisition in one or two heart beats at every heart frequency and body patient’s weight. Therefore, in these cases pretreatment with beta-blockers or ivabradine is not required unless a contextual coronary CT is required. A patient’s narcosis is not routinely required, while sedation can be obtained with short acting benzodiazepine alone or accompanied by ketamine administration. Feed and wrap techniques are also useful for young children in routine contexts [70,71,72,73,74].

## 5. Nuclear Medicine and Marfan Syndrome

Nuclear medicine techniques may represent feasible supplementary diagnostic methods for complications in MFS. Nonetheless, applications of nuclear medicine procedures for the study of complications in Marfan Syndrome are limited to few cases reported in the literature.

In 1993, Yen and Yeh evaluated three patients with MFS and myocarditis, using myocardial single photon emission computed tomography (SPECT) with ^99m^Tc-hexamethylpropyleneamine oxime (^99m^Tc-HMPAO)—labeled white blood cell (WBC) and dypiridamole ^201^Tl: in sites of myocarditis, myocardial SPECT revealed accumulation of ^99m^Tc-HMPAO- labeled WBC and perfusion defects (at ^201^Tl heart scans) [75]. With regard to other possible SPECT applications in MFS, it is important to underline that myocardial SPECT (with ^201^Tl or ^99m^Tc labeled radio compounds such as sestamibi or tetrofosmin) may be useful for the evaluation of functional aspects in cardiomyopathies (such as perfusion, viability, and cardiac pump function), and possible complications in these patients [76].

Regarding future possible scintigraphic applications in patients with Marfan syndrome, an interesting paper by Emrich et al. demonstrated (in a Marfan mouse model) the promising role of SPECT with ^99m^Tc-annexin in the evaluation of increased apoptosis during early aneurysm development in aorta [77].

**Figure 6:**
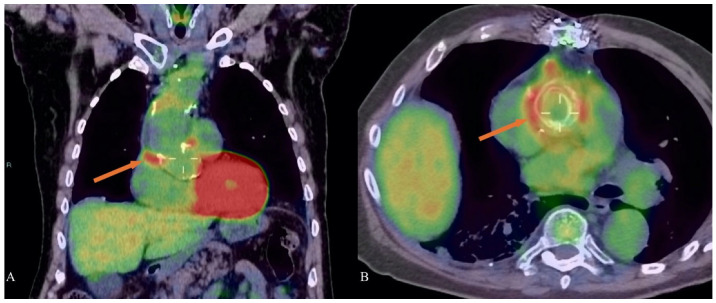
A case of aortic prosthetic graft infection in a patient with Marfan syndrome evaluated with ^18^F-fluorodeoxyglucose (^18^F-FDG) positron emission tomography/computed tomography (PET/CT): coronal (**A**) and axial (**B**) images show hypermetabolism around the aortic prosthetic graft (orange arrows) and site of infection process.

Positron emission tomography/computed tomography (PET/CT) with ^18^F- Fluorodeoxyglucose (^18^F-FDG) is a diagnostic imaging technique generally requested for oncological patients, but it is currently used for the evaluation of variety of inflammatory and infectious conditions as well. The European Association of Nuclear Medicine (EANM) and the Society of Nuclear Medicine and Molecular Imaging (SNMMI) guidelines suggest the application of ^18^F-FDG PET/CT in inflammations and infections [78]. In fact, ^18^F-FDG is an analog of glucose and it is taken up by white blood cells (in particular neutrophils, macrophages, and monocities), recruited in inflammations/infections sites, due to the high levels of GLUT transporters expressed by these cells [79]. Therefore, the utilization of ^18^F-FDG PET/CT in the diagnosis of inflammatory and infectious complications of Marfan syndrome may be useful (Figure 6), as reported by various authors in the literature.

^18^F-FDG PET/CT represent an important tool for the assessment of aortic wall inflammation in these patients, as described in an interesting paper by Brili et al. The authors, evaluating fifteen patients with Marfan syndrome with ^18^F-FDG PET/CT, reported higher arterial target-to-background ratios in these patients compared to the control group, thus demonstrating an aortic wall inflammation state. The authors also measured the serum levels of Interleukin 6, Transforming Growth Factor-b, and Macrophage Colony Stimulating Factor, finding significant differences between patients with Marfan syndrome and the control group in this case too [80]. Regarding the use of ^18^F-FDG PET/CT in the diagnosis of aortic wall inflammations in these patients, Lindsay and collaborators reported the case of a 44-year-old male with Marfan syndrome (the patient had a previous metallic aortic valve and aortic root replacement), with general malaise, weight loss, abdominal and lower-back pain. ^18^F-FDG PET/CT was important to detect a chronic peri-aortitis, represented by a peri-aortic hypermetabolic tissue (this tissue presented an extension from the abdominal aorta to both iliac arteries). A subsequent biopsy of the tissue demonstrated lymphoid infiltrations of T and B cells, plasma cells, and lymphoid follicles [81]. In this context, it is worth mentioning the case reported by Johnston et al. [82], in which the authors described a peri-aortitis in a 34-year-old man with Marfan syndrome (the patient presented fever, weight loss, and lower-back pain). In this case, ^18^F-FDG PET/CT revealed a ^18^F-FDG avid soft tissue around the abdominal aorta, with an extension from the renal hila to aortic bifurcation.

^18^F-FDG PET/CT may be useful for the detection of aortic prosthetic graft infections in patients with Marfan syndrome, as reported by Yamanaka et al. [83]; the authors presented the case of a 30-year-old man with Marfan syndrome, who underwent thoracoabdominal aortic aneurysm repair 9 years before. The patient developed a fever of unknown origin, and an aortic prosthetic graft infection was suspected. ^18^F-FDG PET/CT was important since it revealed four infectious sites (with high uptake of the radiopharmaceutical) around the aortic prosthetic graft; subsequently, the patient underwent graft replacement with a rifampicin-bonded gelatin-impregnated Dacron graft, with no recurrent infection after surgery. In this regard, Shim and collaborators presented two interesting cases of graft infection in patients with Marfan syndrome [84], assessed with ^18^F-FDG PET/CT. In the first case, a 40-year-old man who had a previous aortic replacement, presented an infection from Pseudomonas Aeruginosa. ^18^F-FDG PET/CT was able to detect the graft infection, showing high uptake of the radiopharmaceutical with a maximum standardized uptake value (SUVmax) of 6.8. In the second case, a 12-year-old patient who previously underwent a Bentall procedure, presented low pressure, myalgia, and fever with a suspected infection of the prosthetic graft. ^18^F-FDG PET/CT revealed the source of infection, showing a high uptake of the radiopharmaceutical (SUVmax 8.6) around the aortic graft. A subsequent new Bentall procedure was performed and antibiotics were administrated after the surgery, with no further complication in the follow-up period.

When ^18^F-FDG PET/CT is used in patients with Marfan syndrome, a multimodality imaging approach is often mandatory in the case of the metabolic evaluation of periaortic tissues, in order to reduce possible misinterpretations (it is well known that a variety of tumors are ^18^F-FDG avid). In this regard, an interesting case reported by Chiocchi et al. [85] may explain this important concept. The authors presented the case of an 18-year-old patient with Marfan syndrome, who previously underwent cardiac surgery with Bentall procedure. A CT scan with triphasic acquisition showed a peri-prosthetic tissue that presented radiological features suspicious for a lymphomatous mass. A ^18^F-FDG PET/CT was required to evaluate the potential uptake of this tissue, showing high uptake of the tracer (SUVmax 10.05) and reinforcing the possible lymphoid nature. Subsequent examinations such as cardiac magnetic resonance demonstrated the true of the mass, that was a postoperative pseudoaneurysm.

Other PET radiopharmaceuticals may have a potential role in the detection of inflammation/infectious sites in patients with Marfan syndrome. In this context, WBCs can be labeled with ^18^F-FDG, allowing the evaluation of cardiac implants infections after surgery with promising results [86,87], but further data are needed to assess the feasibility of this diagnostic approach.

^68^Ga-DOTATOC and ^68^Ga-DOTANOC are radiolabeled somatostatin analogs used in PET/CT for the evaluation of well-differentiated neuroendocrine tumors, since these radiopharmaceuticals target somatostatin receptors with high affinity [88]. Activated macrophages and lymphocytes exhibit a high expression of somatostatin receptors as well [86] and PET/CT with ^68^Ga-DOTATOC or ^68^Ga-DOTANOC may have potential applications for the detection of myocardial inflammations (with a possible diagnostic role in patients with Marfan Syndrome), as reported in a few interesting cases [89,90]. The role of radiolabeled somatostatin analogs in the detection of myocardial inflammations is currently under investigation in clinical trials [91]. Other interesting future perspectives may be represented by novel PET radiopharmaceuticals targeting mitochondrial translocator proteins and/or matrix metalloproteinases, promising for the evaluation of disease progression in aortic aneurisms [92].

Table 2 summarizes specific recommendations for the application of various imaging modalities in the diagnosis and management of Marfan Syndrome, aiming to enhance diagnostic precision and patient outcomes.

## 6. Literature Limitations in Multi-Imaging Modalities in Marfan Syndrome

The current literature shows some limitations across imaging modalities in MFS. For instance, echocardiography lacks standardized protocols for Z-score calculations, especially in pediatric populations, leading to inconsistent aortic measurements and complicating clinical decision-making [93].

Similar variability affects advanced techniques like CMR 4D flow and feature-tracking strain, hindering their standardization and broader clinical adoption. The underutilization of strain imaging further delays the early detection of subclinical myocardial dysfunction. While echocardiographic and CMR tools can accurately characterize mitral annular disjunction (MAD), limited research has explored its prevalence, prognostic value, or implications for management strategies

Nuclear medicine techniques, such as ^18^F-FDG PET/CT and myocardial SPECT, remain underexplored in MFS, with evidence primarily based on case reports or small cohorts. The lack of standardized nuclear imaging protocols and their limited feasibility restricts their utility in routine practice. Addressing these gaps through collaborative research, standardized methodologies, and expanded studies will enhance the diagnostic precision, prognostic insights, and clinical applicability of these imaging tools in MFS.

## 7. Conclusions and Future Perspectives

The management of MFS has significantly evolved with advancements in cardiovascular multimodality imaging. Each imaging technique—echocardiography, CMR, CT, and nuclear medicine—offers unique advantages and plays a vital role in the comprehensive care of MFS patients.

Echocardiography remains the cornerstone of initial diagnosis and routine monitoring due to its accessibility and non-invasive nature. It is indispensable for evaluating aortic root dilatation and mitral valve abnormalities, guiding the timing of surgical interventions and assessing the risk of life-threatening complications.

CMR has become a critical tool for detailed anatomical and functional assessment without radiation exposure. It provides comprehensive insights into aortic and myocardial health, enabling the early detection of cardiomyopathy and facilitating personalized management plans. CMR’s ability to evaluate hemodynamic parameters such as pulse wave velocity and aortic distensibility adds a valuable dimension to risk stratification and intervention planning. These features make CMR crucial for a comprehensive evaluation of both aortic and myocardial complications.

CT with its high-resolution imaging capabilities, is essential for precise measurements and detailed visualization of the aorta, particularly in preoperative planning and in cases where echocardiographic windows are inadequate. However, the associated ionizing radiation necessitates judicious use, particularly in younger patients. Recent technological advancements, such as photon-counting CT, can reduce radiation risks while maintaining high-resolution imaging quality.

Nuclear Medicine, though less commonly employed, offers promising applications in evaluating inflammatory and infectious complications in MFS. Techniques such as PET/CT with ^18^F-FDG and myocardial SPECT provide unique insights into metabolic and inflammatory processes, contributing to a more comprehensive understanding of disease complications and aiding in the management of complex cases. Furthermore, novel matrix metalloproteinase PET radiopharmaceuticals could contribute in the future to the assessment of disease progression in aortic aneurysms.

The integration of these imaging modalities into a cohesive diagnostic and monitoring strategy allows for personalized risk stratification and for the prevention of life-threatening complications, optimizing outcomes in MFS patients. Regular surveillance using echocardiography, complemented by periodic CMR assessments, ensures the timely detection of complications and guides therapeutic interventions. In select cases, CT and nuclear medicine techniques provide additional diagnostic information, particularly in preoperative planning and the evaluation of inflammatory conditions.

Looking forward, continued advancements in imaging technologies and techniques will further enhance our ability to manage MFS. The development of novel imaging biomarkers and the integration of artificial intelligence in image analysis hold promise for even more precise and personalized patient care. Multicenter collaborations and longitudinal studies are essential to validate emerging techniques and establish standardized protocols for the comprehensive management of MFS.

In conclusion, cardiovascular multimodality imaging is indispensable in the care of Marfan syndrome patients. By leveraging the strengths of each imaging modality, clinicians can achieve a thorough understanding of disease progression, optimize timing for interventions, and ultimately improve patient outcomes. The future of MFS management lies in the continued evolution of multimodality imaging technologies and the adoption of a multidisciplinary approach to patient care.

## Figures and Tables

**Table 1:** This table provides a concise summary of the criteria used in the 2010 revised Ghent nosology for the diagnosis of Marfan Syndrome. It includes key diagnostic rules based on the presence or absence of family history, along with considerations for differential diagnosis and genetic testing.

Criteria	Diagnosis of Marfan Syndrome	Additional Considerations
In the Absence of Family History		
Aortic Root Dilatation (Z-score ≥ 2) AND Ectopia Lentis	Marfan Syndrome	The presence of aortic root enlargement (Z-score ≥ 2, standardized by age and body size) or dissection with ectopia lentis confirms Marfan Syndrome. Differential diagnoses such as Shprintzen-Goldberg syndrome, Loeys-Dietz syndrome (TGFBR1/2, SMAD3, TGFB2, TGFB3), or vascular Ehlers-Danlos syndrome (COL3A1) must be excluded in the case of the presence of suggestive features.
Aortic Root Dilatation Z score ≥ 2 AND FBN1 Mutation	Marfan Syndrome	Diagnosis is confirmed with aortic root enlargement (Z ≥ 2) or dissection alongside a verified FBN1 mutation, even in the absence of ectopia lentis.
Aortic Root Dilatation Z score ≥ 2 AND Systemic Score ≥ 7 points	Marfan Syndrome	A diagnosis is supported when systemic findings (≥7 points) accompany aortic root enlargement (Z ≥ 2) or dissection. Features indicative of other syndromes (e.g., Shprintzen-Goldberg syndrome, Loeys-Dietz syndrome, or vascular Ehlers-Danlos syndrome) require genetic testing of TGFBR1/2, SMAD3, TGFB2, TGFB3, or COL3A1.
Ectopia Lentis AND FBN1 Mutation associated with Aortic Root Dilatation	Marfan Syndrome	If ectopia lentis is present without aortic enlargement or dissection, an FBN1 mutation linked to aortic disease is required for diagnosis.
In the Presence of Family History		
Ectopia lentis AND Family History of Marfan syndrome (as defined above)	Marfan Syndrome	Diagnosis is established if ectopia lentis is present with a family history meeting the criteria for Marfan Syndrome (as defined in 1–4 above).
A systemic score ≥ 7 points AND Family History of Marfan syndrome (as defined above)	Marfan Syndrome	A systemic score of ≥7 points combined with a family history confirms the diagnosis. Alternative syndromes, such as Loeys-Dietz (TGFBR1/2, SMAD3, TGFB2, TGFB3) or vascular Ehlers-Danlos syndrome (COL3A1), must be excluded via genetic testing in the case of the presence of suggestive features.
Aortic Root Dilatation Z score ≥ 2 above 20 yrs. old, ≥3 below 20 yrs. old + Family History of Marfan syndrome (as defined above)	Marfan Syndrome	For individuals aged >20 years (Z ≥ 2) or ≤20 years (Z ≥ 3) with a relevant family history, the diagnosis is confirmed. Differential diagnoses, including Loeys-Dietz (TGFBR1/2, SMAD3, TGFB2, TGFB3) and vascular Ehlers-Danlos syndrome (COL3A1), must be excluded through appropriate genetic testing in the case of the presence of suggestive features.

**Table 2:** Specific recommendations for different imaging modalities.

Modality	Specific Recommendations
Echocardiography	Regularly evaluate aortic root and ascending aorta dimensions using standardized techniques (e.g., leading-edge-to-leading-edge in adults). Index measurements to body surface area and calculate Z-scores (≥2 indicates concern). Screen for mitral valve prolapse (MVP) and mitral annular disjunction (MAD) and monitor for complications like regurgitation. Include left and right ventricular function assessment, integrating strain imaging for early myocardial dysfunction detection. Monitor pulmonary artery dimensions, especially the root, for potential dilation.
Cardiovascular Magnetic Resonance (CMR)	Use CMR for comprehensive evaluation of the entire aorta, including aneurysms, dissections, and hemodynamic properties (4D flow). Incorporate CMR into routine monitoring to detect disease progression, particularly in younger patients. Report the measurement techniques to ensure consistency in measurements. Use feature-tracking techniques to detect early myocardial dysfunction and evaluate the impact of skeletal deformities on cardiac function, including right ventricular impairment. Use contrast-enhanced imaging to identify arrhythmogenic regions, particularly in the case of MAD.
Computed Tomography (CT)	Use CT for detailed evaluation of the entire aorta, particularly in emergencies like dissections. Perform assessments in multiple phases (non-contrast, arterial, and late) for accurate characterization of calcifications, intramural hematomas, and contrast leaks. Apply dose-reduction techniques to minimize radiation exposure, particularly in pediatric populations. Administer contrast tailored to patient size and condition (e.g., 2 mL/kg in children, 1.5 mL/kg in adults) with appropriate infusion rates for optimal imaging quality. Standardize aortic measurements in diastole, reporting dimensions consistently (e.g., outer-to-outer for thickened walls, inner-to-inner for routine measurements). Employ 3D reconstruction tools for surgical planning and follow-up monitoring. Integrate CT into longitudinal follow-up of aortic enlargement, particularly in cases where echocardiography alone provides inconsistent results.
Nuclear Medicine	Use ^18^F-FDG PET/CT to detect aortic wall inflammation and complications such as chronic peri-aortitis or infections in prosthetic grafts. Utilize myocardial SPECT with ^201^Tl or ^99m^Tc-based tracers for evaluating perfusion, viability, and cardiac pump function in Marfan cardiomyopathy, particularly when echocardiographic or CMR data are inconclusive.

## Abbreviations

AD	Aortic Distensibility
BAV	Bicuspid Aortic Valve
b-SSFP	Balanced Steady-State Free Precession
BSA	Body Surface Area
CCT	Cardiac Computed Tomography
CEMRA	Contrast-Enhanced Magnetic Resonance Angiography
CMR	Cardiovascular Magnetic Resonance
EF	Ejection Fraction
EANM	European Association of Nuclear Medicine
ESC	European Society of Cardiology
GLS	Global Systolic Longitudinal Strain
HI	Haller Index
HU	Hounsfield Units
LGE	Late Gadolinium Enhancement
LV	Left Ventricle
LVEDD	Left Ventricular End-Diastolic Diameter
MAD	Mitral Annular Disjunction
MFS	Marfan Syndrome
MR	Mitral Regurgitation
MRI	Magnetic Resonance Imaging
MVP	Mitral Valve Prolapse
PC	Photon Counting
PET	Positron Emission Tomography
PSI	Pixel Signal Intensity
PWV	Pulse Wave Velocity
ROI	Region of Interest
RV	Right Ventricle
SPECT	Single Photon Emission Computed Tomography
SNMMI	Society of Nuclear Medicine and Molecular Imaging
SSFP	Steady-State Free Precession Imaging
TAPSE	Tricuspid Annular Plane Systolic Excursion
TDI	Tissue Doppler Imaging
TEE	Transesophageal Echocardiography
TTE	Transthoracic Echocardiography
VT	Ventricular Tachycardia
WSS	Wall Shear Stress
^99m^Tc-HMPAO	Technetium-99m hexamethylpropyleneamine oxime
WBC	White Blood Cell Introduction
